# The effects of different forms of daily exercise on metabolic function following short-term overfeeding and reduced physical activity in healthy young men: study protocol for a randomised controlled trial

**DOI:** 10.1186/s13063-018-2579-6

**Published:** 2018-03-27

**Authors:** Jean-Philippe Walhin, Yung-Chih Chen, Aaron Hengist, James Bilzon, James A. Betts, Dylan Thompson

**Affiliations:** Human Physiology Research Group, Department for Health, University of Bath, Bath, BA2 7AY UK

**Keywords:** Exercise, Overfeeding, Reduced physical activity, Arm crank, Breaking sitting, Moderate-intensity exercise, Metabolism, Adipose tissue, Skeletal muscle and energy imbalance

## Abstract

**Background:**

Short-term overfeeding combined with reduced physical activity impairs metabolic function and alters the expression of key genes within adipose tissue. We have shown that daily vigorous-intensity running can prevent these changes independent of any net effect on energy imbalance. However, which type, intensity and/or duration of exercise best achieves these benefits remains to be ascertained.

**Methods/design:**

Forty-eight healthy young men will be recruited and randomly allocated to one of four experimental conditions for 1 week: (1) to ingest 50% more energy than normal by over-consuming their habitual diet whilst simultaneously restricting their physical activity below 4000 steps day^−1^ (i.e. energy surplus; SUR group); (2) the same regimen but with a daily 45-min bout of vigorous-intensity arm crank ergometry at 70% of maximum oxygen uptake (SUR + ARM group); (3) the same regimen but with a daily 45-min bout of moderate-intensity treadmill walking at 50% of maximum oxygen uptake (SUR + MOD group); (4) the same regimen but with the addition of intermittent short bouts of walking during waking hours (SUR + BREAKS group). Critically, all exercise groups will receive additional dietary energy intake to account for the energy expended by exercise, thus maintaining a matched energy surplus. At baseline and follow-up, fasted blood samples, abdominal subcutaneous adipose tissue and skeletal muscle biopsies will be obtained and oral glucose tolerance tests conducted.

**Discussion:**

This study will establish the impact of different forms of daily exercise on metabolic function at the whole-body level as well as within adipose tissue and skeletal muscle in the context of a standardised energy surplus.

**Trial registration:**

ISRCTN, ISRCTN18311163. Registered on 24 June 2015.

**Electronic supplementary material:**

The online version of this article (10.1186/s13063-018-2579-6) contains supplementary material, which is available to authorized users.

## Background

It is generally accepted that regular physical activity/exercise is an important part of a healthy lifestyle and that physical inactivity increases the risk of disease over the long term [1]. Both an acute bout of exercise and chronic endurance training can have beneficial effects on outcomes that are important for health, such as insulin action. Earlier work by Hagobian and Braun [2] reported rapid metabolic dysregulation following 3 days of overfeeding without structured exercise in a cohort of habitually active participants. Importantly, however, a single bout of moderate-intensity exercise restored insulin responses to baseline despite additional overfeeding on the final day to match energy expended during exercise. This finding presents the interesting possibility that exercise has the potential to impact metabolism independent of any effect on energy balance. In order to understand and isolate how exercise exerts its effects on metabolism, it is critically important to conduct studies where energy status is carefully controlled [3]. Human models where physical activity/exercise and energy balance are manipulated offer an opportunity to tease apart the impact of exercise above and beyond its impact on energy balance and/or adiposity. One of the most powerful ways to examine this question is through a deliberate attempt to create an energy surplus with and without various forms of physical activity.

Various studies have investigated the impact of reduced physical activity on health outcomes in healthy, active individuals. Both short-term and longer-term physical inactivity, induced by restricting structured exercise [4, 5] or by reducing the daily number of steps [6, 7], have consistently led to a decrease in insulin sensitivity. More extreme bed-rest studies have also shown how complete physical inactivity can reduce insulin sensitivity and alter the skeletal muscle transcriptome [8–11]. Numerous studies have investigated the impact of positive energy balance induced through overfeeding of varied duration and composition on metabolic outcomes. Excessive energy intake impairs insulin sensitivity [12–14] and alters adipose gene expression [15–17]. Many metabolic diseases such as type 2 diabetes, obesity and the metabolic syndrome result from a chronic state of energy imbalance created by low physical activity and/or an excessive energy intake. It is remarkable that very few studies have used experimental models to investigate the joint impact of both factors applied concurrently.

The combination of overfeeding and decreased physical activity can impair glycaemic control and insulin sensitivity [18–20]. Our previous work has shown that short-term overfeeding combined with reduced physical activity induces a state of insulin resistance, hyperinsulinaemia and altered expression of key genes within adipose tissue [20]. The addition of daily *vigorous-intensity running* mostly prevented these changes independent of any net effect on energy imbalance. Whilst running involves a large muscle mass and has the potential to expend many calories (potentially providing a route of disposal for excess glucose), it is unknown whether other forms of physical activity have the same impact. Alternative forms of exercise may be more appropriate for or preferred by specific populations.

The objectives of the proposed study are to evaluate three alternative but energy-matched physical activity/exercise models:

1. To establish whether a daily bout of *vigorous-intensity upper-body exercise* can prevent negative changes in metabolic function induced by short-term overfeeding and reduced physical activity
2. To establish whether a daily bout of *moderate-intensity treadmill walking* can prevent negative changes in metabolic function induced by short-term overfeeding and reduced physical activity
3. To establish whether *several short bouts of walking* throughout the day can prevent negative changes in metabolic function induced by short-term overfeeding and reduced physical activity

## Methods/design

### Participants

To address these objectives, a cohort of 48 healthy young men who satisfy the inclusion and exclusion criteria outlined below will be recruited via advertisement from the local community. Written consent will be obtained from all participants after the initial meeting, and participants will be informed that they can withdraw from the study at any time without consequence. Eligibility will be assessed by a series of self-report questionnaires. Eligible participants will then be randomly allocated to one of four parallel intervention arms using a stratified randomisation scheme. The randomisation schedule will be generated by a senior author (JBe) using an electronic random number generator and concealed from those involved in participant management (JPW) to prevent biased allocation [21, 22]. Participant assignment will be requested via email, and full details of the overall randomisation scheme will be published once all allocations are completed to prevent deciphering. This will feature a factor for objectively measured physical activity level (PAL) and body mass to ensure an even distribution of less active (PAL: <1.75) and more active (PAL: ≥1.75) as well as lighter (<77 kg) and heavier (≥77 kg) participants in each treatment arm, respectively. The ensuing protocol for this study has received ethical approval from the National Health Service Research Ethics Committee (reference: 15/SW/0014).

Inclusion:

1. Male.
2. Aged 18–40 years old.
3. Active (exercise more than 30 min per day, at least three times per week).
4. Stable body mass for at least 6 months (<3% increase or decrease [23]).
5. Able and willing to comply with study procedures.
6. Have the capacity to provide informed consent.

Exclusion:

1. Female.
2. Currently smoking.
3. Aged less than 18 years or greater than 40 years.
4. Positive response to one or several questions from the Physical Activity Readiness Questionnaire (PAR-Q).
5. Taking any medication that might interfere with the study outcomes (This will be reviewed by the research team on a case-by-case basis should a potential participant be on regular medication. The British National Formulary will be checked for potential effects that might introduce bias in the study.).
6. Illness/condition that might interact with study measures (e.g. diabetes, heart disease) or pose undue personal risk.

### Power calculation

We plan to recruit 48 young (18–40 years), healthy, weight-stable, active men from the local population. Using data from our previous study [20], it was estimated that a sample size of 12 in each treatment group will provide approximately 90% power to detect a difference in ∆ insulin iAUC of 17 ± 16 versus 1 ± 6 nmol 120 min L^−1^ using an independent *t* test at an alpha level of 0.05. Recruitment will proceed on a rolling basis until the adequate sample size is reached; emphasis will be placed on considering the demands of the study before enrolling to reduce loss to follow-up.

### Statistical analysis

All data from participants having successfully completed the study will be included in the analysis. The statistical analysis plan is for a primary comparison between intervention groups and control arm. The precise time-course of responses within and between trials will be analysed using factorial two- and three-way mixed-model analysis of variance (group × day and group × day × time, respectively). For all the above statistical approaches, statistical significance will be set at an alpha level of *p* ≤ 0.05. We will explore the use of confidence intervals and magnitude-based inferences to assess the clinical significance of the effect.

### Experimental design and protocol description

A randomised parallel group design will be used for this trial (registration number: ISRCTN18311163). Participants will be randomly allocated by a third party to experience either:

1. A fixed energy surplus via 7 days of overfeeding (+50%) and residual physical activity (≤4000 steps day^−1^) (SUR)

OR

2. A matched energy surplus and residual physical activity (≤4000 steps day^−1^) with a daily 45-min bout of vigorous-intensity arm crank ergometry (SUR + ARM)

OR

3. A matched energy surplus and residual physical activity (≤4000 steps day^−1^) with a daily 45-min bout of moderate-intensity treadmill walking (SUR + MOD)

OR

4. A matched energy surplus with the addition of intermittent short bouts of walking during waking hours equivalent to the energy that would be expended by each participant during exercise if they had been allocated to the SUR + MOD group with an additional allowance of 4000 steps day^−1^ to account for permissible residual physical activity as in the other groups (SUR + BREAKS)

#### SUR group

In the SUR group, participants will restrict their daily physical activity to ≤4000 steps per day with no structured exercise allowed and will be required to over-consume their habitual diet by 50%.

#### SUR + ARM group

**Fig. 1:**
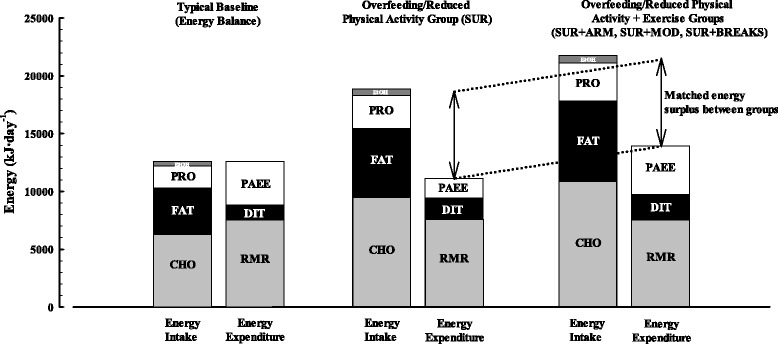
Schematic representation of the planned energy surplus that will be induced by the overfeeding and restricted physical activity models. The energy surpluses will be matched between all groups. CHO carbohydrates, PRO protein, EtOH alcohol, RMR resting metabolic rate, DIT diet-induced thermogenesis, PAEE physical activity energy expenditure

In the SUR + ARM group, participants will also restrict their daily physical activity to ≤4000 steps per day with no structured exercise allowed except a daily 45-min bout of arm crank ergometry at 70% V̇O_2peak_. Importantly, the SUR + ARM group will be prescribed additional energy intake to account for the energy expended during the exercise (i.e. habitual energy intake will be increased by 50% + energy expended during the exercise in order to standardise energy surplus; Table 1 and Fig. 1). The first exercise bout in the SUR + ARM group will be supervised, and compliance to the following six exercise bouts will be confirmed by combined heart rate/accelerometry (Actiheart, Cambridge Neurotechnology Ltd., Cambridge, UK) by reviewing each participant’s physical activity records after they have completed the study. The final exercise bout prescribed under the SUR + ARM treatment will be performed at a standardised time of day specific to each participant. This will take place at the same time as the exercise bout each individual had performed the day before baseline measures were taken and before 1400 h (i.e. to facilitate meaningful comparisons between baseline and follow-up).

**Table 1:** Intervention arms employed in the study protocol

Intervention	Description
SUR	Overfeeding (50%) and restricted physical activity (≤4000 steps day^−1^)
SUR + ARM	Overfeeding (50% + energy expended during exercise) and restricted physical activity (≤4000 steps day^−1^) with a daily bout of vigorous-intensity arm crank ergometry (45 -min at 70% V̇O_2peak_)
SUR + MOD	Overfeeding (50% + energy expended during exercise) and restricted physical activity (≤4000 steps day^−1^) with a daily bout of moderate-intensity treadmill walking (45 -min at 50% V̇O_2max_)
SUR + BREAKS	Overfeeding (50% + energy expended during walking bouts) with addition of intermittent short bouts of walking during waking hours equivalent to the energy that would be expended by each participant during exercise if they had been allocated to the SUR + MOD group with an additional allowance of 4000 steps day^−1^to account for permissible residual physical activity in the other groups

#### SUR + MOD group

In the SUR + MOD group, participants will also restrict their daily physical activity to ≤4000 steps per day with no structured exercise allowed except a daily 45-min bout of treadmill walking at 50% maximal oxygen uptake (V̇O_2max_). Importantly, the SUR + MOD group will be prescribed additional energy intake to account for the energy expended during the exercise (i.e. habitual energy intake will be increased by 50% + energy expended during the exercise in order to standardise energy surplus; Table 1 and Fig. 1). The first exercise bout in the SUR + MOD group will also be supervised, and compliance during the intervention will also be confirmed by combined heart rate/accelerometry records (Actiheart, Cambridge Neurotechnology Ltd., Cambridge, UK). The final exercise bout prescribed under the SUR + MOD treatment will be performed at a standardised time of day specific to each participant as in the SUR + ARM group.

#### SUR + BREAKS group

In the SUR + BREAKS group, participants will be asked to perform small bouts of walking throughout the day to reach their individual target steps with no structured exercise allowed. The daily target steps will be calculated from the data collected during the self-selected speed test and target steps calculation procedure. The energy expended during the walking bouts will be equivalent to the energy expended if each participant had been allocated to the SUR + MOD group, with an additional allowance of 4000 steps each day to account for permissible residual physical activity as in the other groups (i.e. the other groups will be allowed to do up to 4000 steps a day). Importantly, the SUR + BREAKS group will be prescribed additional energy intake to account for the energy expended during walking (i.e. habitual energy intake will be increased by 50% + energy expended during the walking bouts in order to standardise energy surplus; Table 1 and Fig. 1). Compliance to the walking bouts in the SUR + BREAKS group will also be confirmed by combined heart rate/accelerometry records (Actiheart, Cambridge Neurotechnology Ltd., Cambridge, UK). Participants will be advised to walk “little and often” throughout the day (i.e. approximately every 30–40 min), each bout lasting ~5 min (approximately 500–600 steps). In addition, participants will be asked to limit longer walks of greater than 1500 steps to only once per day, and to try and not exceed a maximum of 2000 steps in a single bout. Participants will also be instructed not to sit for longer than 1 h at a time.

### Calculations of energy surplus

The excess post-exercise oxygen consumption (EPOC) associated with each exercise bout will be estimated as 8.5%, 5.2% and 5% of energy expended during the exercise for the SUR + ARM [24], SUR + MOD [25] and SUR + BREAKS [25] groups, respectively. The diet-induced thermogenesis (DIT) associated with the extra food prescribed to all three intervention groups (compared to the SUR group) will be calculated as 10% of the energy expended during the bout of exercise (including EPOC [26]). The estimated EPOC and DIT associated with the exercise bout will then be added to the prescribed energy intake. Finally, the contribution of resting metabolic rate (RMR) towards total energy expenditure during each exercise session (45 min or equivalent for the SUR + BREAKS group) will be subtracted from the overfeeding calculation as this will have been already taken into account when prescribing the 50% overfeed based on a 24-h RMR.

### Preliminary assessments

#### Diet and physical activity assessments

Over a period of seven consecutive days, participants will record their habitual food and fluid intake prior to taking part in the study using a set of digital weighing scales (Model 3001, Salter, Kent, UK). Dietary records will be analysed using the Nutrition Analysis Software version 1.8 (Nutritics, UK), which is based on a food database in the UK. Energy intake will be estimated using this software, and DIT will be estimated pre-intervention as 10% of energy intake [26]. During this period, a combined heart rate/accelerometer monitor (Actiheart, Cambridge Neurotechnology Ltd., Cambridge, UK) will be used to determine habitual physical activity thermogenesis at baseline. A pedometer (3D TriSport, China) will be worn to record habitual daily step count.

#### Pre-trial fitness tests

In order to avoid unnecessary participant burden, maximum oxygen uptake will be assessed on the treadmill *and* on the arm crank ergometer for participants in the SUR group, but participants in the three exercise groups will only be required to have their relevant maximum oxygen uptake and other tests assessed (either on the treadmill or on the cycle ergometer).

##### Treadmill V̇O_2max_

Running maximal oxygen uptake (V̇O_2max_) tests will be performed by participants from the SUR, SUR + MOD and SUR + BREAKS groups at least 1 week prior to the first main trial. Participants’ V̇O_2max_ will be assessed on a treadmill prior to the intervention (ELG70, Woodway, Weiss, Germany) following a method adapted from Taylor et al. [27]. The percentage of O_2_ and CO_2_ in expired air samples for all tests will be determined using paramagnetic and infrared gas analysers, respectively (Series 1400, Servomex Ltd., Sussex, UK), calibrated according to the manufacturer’s instructions prior to use.

##### Arm crank V̇O_2peak_

Peak oxygen uptake (V̇O_2_ peak) will be determined for participants from the SUR and SUR + ARM groups using a continuous, progressive intensity test on an electrically braked arm crank ergometer (Lode Angio, Groningen, Netherlands). A cadence of 75 rpm will be encouraged throughout, and a starting intensity will be selected based on the participant’s ability. The resistance will be increased by 14 W every 3 min until the point of volitional exhaustion (approximately 9–12 min).

##### Gradient walking test

A gradient walking test will be performed by participants from the SUR + MOD and SUR + BREAKS groups to determine their 50% V̇O_2max_. The gradient walking test will be undertaken on a treadmill (ELG70, Woodway, Weiss, Germany) maintained at 6 to 7 km h^−1^ with an initial 2.5% gradient that will be increased by 2.5% every 5 min until the gradient reached 10%. To determine energy expenditure, expired air samples will be collected for 1 min at the end of each 5-min stage.

##### Self-selected speed test and target steps calculation

Participants randomised in the SUR + BREAKS group will perform a 15-min walking test at a self-selected speed to estimate the number of steps achieved in that time. Participants will be allowed 1 min to choose their “normal walking speed” whilst blinded from the treadmill speedometer. Once the speed has been selected, participants will walk at the chosen speed for the remaining 14 min, and expired air will be collected for 1 min at 5-min intervals to determine energy expenditure [28] and the total number of steps will be recorded during that time. The number of steps required to expend the equivalent energy as a 45-min walking bout at 50% V̇O_2max_ will be determined for each participant. Participants will then be assigned a specific number of steps to complete each day of the intervention period to match the energy expended during exercise if they had been allocated to the SUR + MOD group. This will be achieved by intermittent short bouts of walking during waking hours in the SUR + BREAKS group instead of a single bout for the SUR + MOD group and an additional allowance of 4000 steps day^−1^ to account for permissible residual physical activity as in the other groups. To monitor this, pedometers will be worn each day during the intervention period to record the total step count. Pedometers will be worn by all other groups to measure adherence to the permitted 4000 steps day^−1^.

### Main trial day protocol

**Fig. 2:**
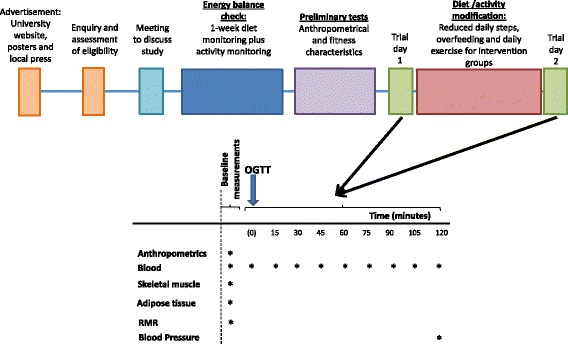
Overview of trial outline, including all measurements and sampling that will take place

**Fig. 3:**
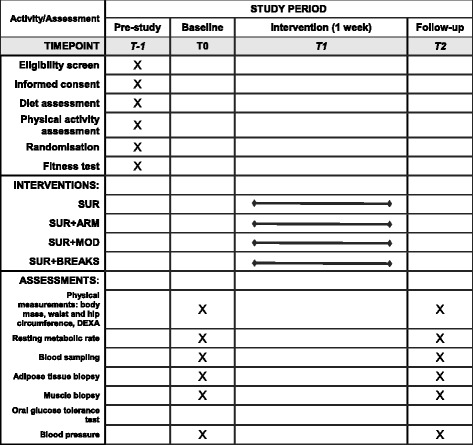
SPIRIT figure showing an overview of the assessment schedule at baseline and follow-up in the study. DEXA dual-energy X-ray absorptiometry

The same experimental procedures will be completed on both baseline and follow-up trial days. During 3 days leading up to the intervention week, volunteers will be asked to adhere to their habitual lifestyle (i.e. diet and physical activity/exercise), and all participants will be asked to perform a 30-min bout of running the day before starting the intervention before 1400 h at their usual running pace. This will ensure that the last bout of exercise will have been standardised for all participants. On the morning of the baseline trial (Fig. 2), participants will report to the laboratory at 0700 ± 0.5 h following an overnight fast (≥10 h). Twenty-four hours prior to each main laboratory trial day, participants will abstain from caffeine (tea/coffee) and alcohol. The SPIRIT figure showing the respective time points for assessments and intervention is provided in Fig. 3 (see Additional file 1).

### Body composition analysis

Body mass will be assessed to the nearest 0.1 kg using digital scales (TANITA Corp., Tokyo, Japan) with participants wearing identical light clothing at baseline and follow-up. Waist and hip circumference will be measured using a non-stretch tape to the nearest 0.1 cm, based on World Health Organization guidelines [29]. Body composition will be assessed by dual-energy X-ray absorptiometry (DEXA; Discovery, Hologic, Bedford, UK) to determine muscle mass and fat mass. All measurements will be made following a quality control scan of a spine phantom with known properties in accordance with the manufacturer’s instructions.

### Resting metabolic rate measurement

Participants will then rest in bed for 10 min, after which resting metabolic rate (RMR) will be determined via indirect calorimetry based on four 5-min expired gas collections with concurrent measurement of inspired air composition; a reliance of standard atmospheric concentrations has recently been discouraged [30]. RMR will be accepted as stable within 100 kcal day^−1^ [31, 32], and the lowest of these measures will be accepted as RMR [20].

### Blood sampling

Following RMR assessments, a 20-G cannula (BD Venflon™ Pro, Becton, Dickinson & Co., Sweden) will be inserted into an antecubital vein from which baseline 20 mL of blood will be drawn through two 10-mL syringes and placed into serum separation beads and EDTA-containing tubes (Sarstedt Ltd, Leicester, UK). Plasma samples will be centrifuged immediately at 3466*g* at 4 °C for 10 min (Heraeus Biofuge Primo R, Kendro Laboratory Products Plc., Tyne and Wear, UK). Serum samples will be left to clot for 15 min at room temperature before centrifugation. All samples will be dispensed into 0.5-mL aliquots and immediately cooled on dry ice and then stored at −80 °C. A small aliquot of EDTA blood will be used to obtain the full leucocyte differential and other haematological variables (SF-300, Sysmex Ltd., Milton Keynes, UK).

### Adipose tissue biopsy and processing

Adipose tissue samples (~0.5 g) will be taken under local anaesthetic (1% lidocaine) from the area around the waist approximately 5 cm lateral to the umbilicus using a 14-G needle using an aspiration technique with follow-up biopsies sampled from the opposite side [20]. The sample will be cleaned with isotonic saline, and any clot will be manually removed. After weighing the sample, it will be homogenised in 5 mL of TRIzol (Invitrogen, UK) and placed on dry ice before being stored at −80 °C.

### Muscle biopsy and processing

After the baseline blood sample and adipose tissue biopsy, a muscle biopsy will then be collected from the vastus lateralis. Participants will rest in a supine position whilst a 3–5-mm skin incision will be made under local anaesthetic (1% lidocaine) using a surgical blade. The percutaneous needle biopsy technique [33] will then be used to obtain between 30 and 100 mg of wet muscle tissue from this site. At follow-up, a muscle biopsy will be taken from each participant’s opposite leg and the use of dominant/non-dominant limbs will be counterbalanced. Muscle samples will be immediately placed in a ventilated Eppendorf tube and snap-frozen in liquid nitrogen where they will be stored during the trial before being stored at −80 °C.

### Oral glucose tolerance test

After the baseline blood sample, adipose tissue and muscle biopsies, participants will consume 113 mL of Polycal (Nutricia Advanced Medical Nutrition, Trowbridge, UK) and 87 mL of water, equivalent to 75 g of anhydrous glucose, within 5 min. Further 5-mL blood samples will be drawn at 15-min intervals for the next 2 h. The intravenous cannula is to be kept patent through periodic flushing with 0.9% NaCl (B. Braun, Sheffield, UK) infusion, with the first 5 mL of each blood drawn being discarded. Blood pressure will also be measured three times during the final 15 min of the oral glucose tolerance test (OGTT) using an automated blood pressure monitor (Boso Medicus Prestige, Bosch + Sohn GmbH, Jungingen, Germany), and the average will be reported.

## Discussion

Our previous work showed that short-term overfeeding combined with reduced physical activity induces a state of insulin resistance, hyperinsulinaemia and altered expression of several key genes within adipose tissue [20]. Importantly, the addition of daily vigorous-intensity running mostly prevented these changes independent of any net effect on energy imbalance [20]. The three exercise models in the present study will enable us to compare the benefits of different physical activity/exercise approaches in the context of increased caloric intake and reduced physical activity. Specifically, the comparison of the SUR + ARM group versus the SUR group will help us establish whether vigorous upper-body exercise has the ability to prevent negative changes in metabolic function induced by short-term overfeeding and reduced physical activity, which may be relevant to wheelchair users. Furthermore, the SUR + MOD and SUR + BREAKS groups will allow us to determine whether moderate-intensity exercise has a similar impact on metabolic health as vigorous-intensity running and whether the same benefits can be gained from distributing that amount of exercise throughout the day. These alternative forms of exercise may be more appropriate for or preferred by specific populations. This study will provide a novel insight regarding the potential of different forms of daily exercise at preserving metabolic function at the whole-body level as well as within adipose tissue and skeletal muscle in the face of a standardised energy surplus. Our analysis will focus on genes and proteins known to be significantly impacted by a week of overfeeding and/or reduced physical activity within both tissues. This approach will help elucidate the mechanisms by which exercise benefits physiological function and human health even in the face of a considerable energy surplus.

## Trial status

Protocol version 1 dated 13 November 2014. Recruitment start date: 01 May 2015. Recruitment end date: 31 August 2017.

## Additional file

Additional file 1: SPIRIT 2013 Checklist: recommended items to address in a clinical trial protocol and related documents. (DOC 121 kb)

## Abbreviations

DEXA: Dual-energy X-ray absorptiometry
DIT: Diet-induced thermogenesis
EDTA: Ethylenediaminetetraacetic acid
EPOC: Excess post-exercise oxygen consumption
iAUC: Incremental area under the curve
OGTT: Oral glucose tolerance test
PAEE: Physical activity energy expenditure
PAL: Physical activity level
PAR-Q: Physical Activity Readiness Questionnaire
RMR: Resting metabolic rate
